# Beyond Empiricism: Diagnostic Value of Kidney Biopsy in Methicillin-Resistant Staphylococcus aureus Endocarditis-Associated Acute Kidney Injury

**DOI:** 10.7759/cureus.96599

**Published:** 2025-11-11

**Authors:** Sirin M Sevil Karapinar, Baris Afsar, Nicolas Mc Donald, Kunal Malhotra, Krista L Lentine

**Affiliations:** 1 Department of Nephrology, Sancaktepe Şehit Prof. Dr. İlhan Varank Training and Research Hospital, Istanbul, TUR; 2 Department of Internal Medicine, SSM (Sisters of Saint Mary) Health Saint Louis University Hospital, St. Louis, USA; 3 Transplant Center, SSM (Sisters of Saint Mary) Health Saint Louis University Hospital, St. Louis, USA

**Keywords:** complement pathway, glomerulonephritis, immune complex formation, infective endocarditis, staphylococcus aureus infection

## Abstract

As intravenous drug use increases, the incidence of right-sided infective endocarditis has also risen. Renal involvement is not uncommon in infective endocarditis, and treatment is often initiated empirically. However, in complex cases with multiple differential diagnoses, kidney biopsy remains the gold standard for establishing the underlying pathology. We report the case of a 35-year-old man with acute kidney injury in the setting of methicillin-resistant *Staphylococcus aureus* infective endocarditis related to intravenous drug use, untreated hepatitis C infection, and possible antibiotic-associated nephrotoxicity. To differentiate these potential causes, a renal biopsy was performed during active infection, providing direct histological evidence of immune complex-mediated glomerulonephritis.

## Introduction

Infection-related glomerulonephritis (IRGN) is an immune complex-mediated disease characterized by significant complement activation [1]. Poststreptococcal glomerulonephritis (PSGN) is the classic prototype, but the spectrum of infections leading to glomerular injury is broad and includes a range of bacterial and viral pathogens [2]. Over recent decades, IRGN has become increasingly prevalent among adults and is now more often associated with non-streptococcal organisms, particularly *Staphylococcus* species. Indeed, staphylococcal IRGN has surpassed poststreptococcal IRGN in prevalence [3].

The World Health Organization (WHO) estimates approximately 470,000 new cases of IRGN annually, with 97% occurring in regions of low socioeconomic status [4,5]. Globally, IRGN remains the leading cause of acute nephritis in children, particularly in developing countries [3]. In contrast, adult IRGN typically occurs in individuals with comorbid conditions such as diabetes mellitus, alcoholism, malignancy, or immunosuppression [6]. Among younger adults, intravenous drug use (IVDU) is an important predisposing factor, often associated with infective endocarditis [7].

Diagnostic criteria for IRGN include at least three of the following: (i) clinical or laboratory evidence of infection preceding or concurrent with glomerulonephritis, (ii) low serum complement levels, (iii) endocapillary proliferative and exudative glomerulonephritis, (iv) C3-dominant or co-dominant glomerular immunofluorescence staining, and (v) hump-shaped subepithelial deposits on electron microscopy [7]. A kidney biopsy is valuable when infection is not well documented, renal function continues to decline, or coexisting conditions complicate the diagnosis.

Alternative diagnoses should be considered when systemic symptoms persist, serum creatinine continues to rise, or hypocomplementemia fails to normalize despite effective infection control [2]. The differential diagnosis includes IgA nephropathy or vasculitis, antineutrophil cytoplasmic antibodies (ANCA)-associated glomerulonephritis, C3 glomerulopathy, and cryoglobulinemic glomerulonephritis [3]. Management focuses on the identification and treatment of the underlying infection with culture-guided antibiotics and supportive care [8]. In adults, unlike in children, who are generally otherwise healthy, comorbid conditions often complicate presentation, supporting the utility of histologic confirmation to guide therapy.

Herein, we report the case of a 35-year-old man with a history of intravenous drug use who developed acute kidney injury (AKI) during treatment for methicillin-resistant *Staphylococcus aureus* (MRSA) tricuspid valve endocarditis, vertebral osteomyelitis, and bacteremia. His comorbidities included untreated hepatitis C virus (HCV) infection. His serum creatinine began rising on day 3 of vancomycin therapy and progressed thereafter. This case illustrates the diagnostic complexity of IRGN in the context of MRSA infection and chronic HCV, where multiple potential causes of renal injury coexist.

## Case presentation

**Figure 1:**
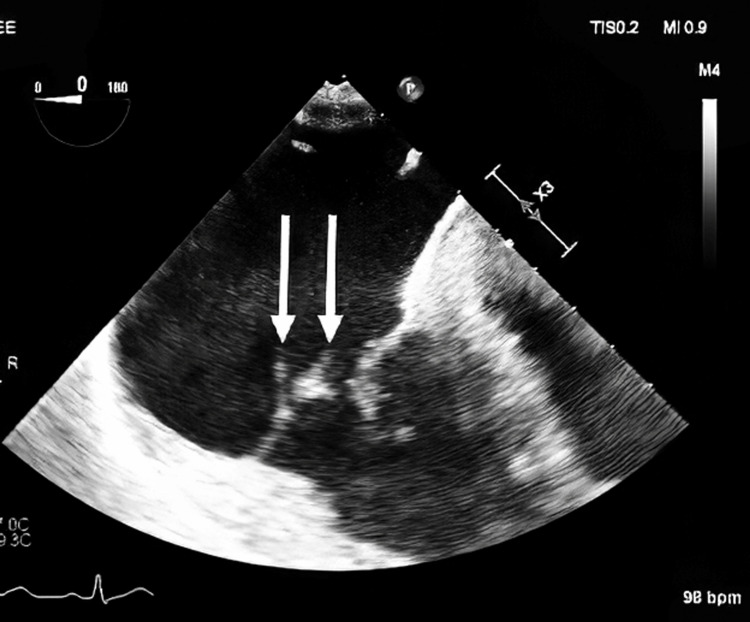
Transthoracic echocardiography demonstrating tricuspid vegetation (white arrows) Echocardiogram demonstrates a large vegetation on the anterior septal leaflet of the tricuspid valve

**Figure 2:**
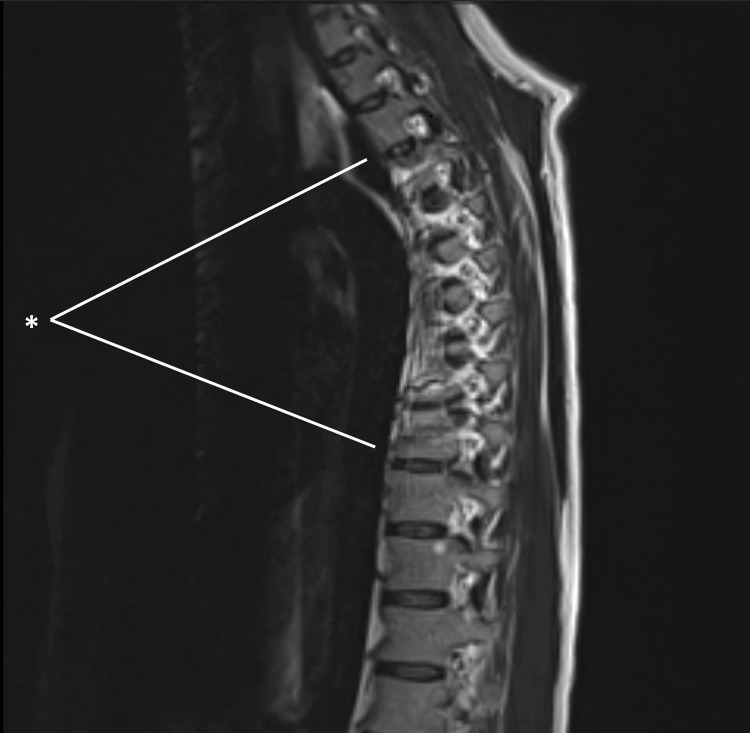
MRI demontrating osteomyelitis. Markers indicate T4-T10 osteomyelitis. The MRI demonstrates an irregular area of hyperintensity within the thoracic spinal cord extending from the T4 to T10 levels. The central canal remains patent, and no neural foraminal stenosis is identified.

**Figure 3:**
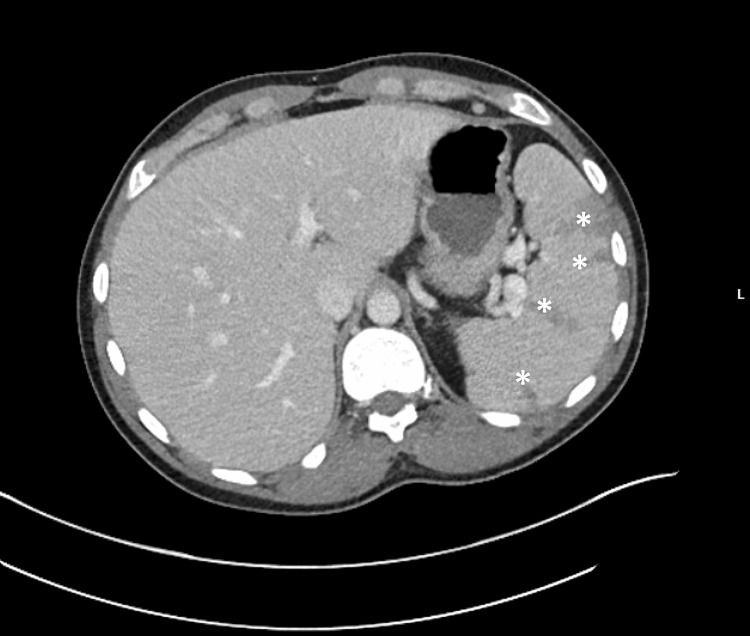
CT abdomen demonstrating splenic infarcts (asterisks). CT shows multiple wedge-shaped areas of decreased enhancement in the spleen, consistent with splenic infarcts.

A 35-year-old Caucasian man with untreated hepatitis C and a history of IVDU presented with fever, chest and back pain, and progressive weakness. He had previously left against medical advice during two admissions for MRSA bacteremia, during which presentation included cavitary lung lesions and transthoracic echocardiography revealed a tricuspid vegetation (Figure 1), magnetic resonance imaging (MRI) showed T4-T10 osteomyelitis (Figure 2), and computed tomography (CT) showed splenic infarcts (Figure 3).

On admission, the patient was febrile and had a right-sided systolic murmur. Kidney function was initially normal (serum creatinine 0.87 mg/dL) but progressively increased to 3.26 mg/dL by hospitalization day 12. Baseline laboratory testing revealed leukocytosis and elevated inflammatory markers, including C-reactive protein (28.3 mg/L) and erythrocyte sedimentation rate (86 mm/h) (Table 1). Urinalysis showed hematuria (3+ blood, >100 RBCs/high-power field), 1+ proteinuria, red blood cell casts, and rare urinary eosinophils; the urine protein-to-creatinine ratio (UPCR) was 1.1 g/g. Complement testing demonstrated low C3 (62 mg/dL; reference range 82-193) with normal C4.

**Table 1:** Baseline laboratory results at current hospitalization

Parameter	Patient Value	Reference Range
White Blood Cell Count	11,600 /L	4,000–10,700 / L
Hemoglobin	9.6 g/dL	13.3–17.5 g/dL
Platelet Count	156,000 /L	150,000–420,000 /L
Sodium	132 mmol/L	136–145 mmol/L
Potassium	3.5 mmol/L	3.5–5 mmol/L
C02	23 mmol/L	22–29 mmol/L
Blood Urea Nitrogen	14 mg/dL	7–26 mg/dL
Creatinine	0.87 mg/dL	0.71–1.16 mg/dL
Calcium	9.4 mg/dL	8.4–10.2 mg/dL
Phosphorus	5.1 mg/dL	2.8–5.1 mg/dL
Magnesium	1.8 mg/dL	1.6–2.6 mg/dL
Alanine aminotransferase	22 U/L	0–55 U/L
Aspartate aminotransferase	20 U/L	5–34 U/L
Albumin	2.4 g/dL	3.4–5 U/L
C-reactive protein	28.3 mg/dL	<=0.5 mg/dL
Erythrocyte sedimentation rate	116 mm/hr	0-15 mm/hr

Blood cultures again grew MRSA, and the patient was started on intravenous vancomycin and ceftaroline (the latter was later discontinued). Vancomycin trough levels were elevated at 30.6 mg/L and 32.5 mg/L on the fifth and eighth days of therapy, respectively. Transesophageal echocardiography confirmed a large tricuspid valve vegetation, while renal ultrasound showed kidneys of normal size and echogenicity.

**Figure 4:**
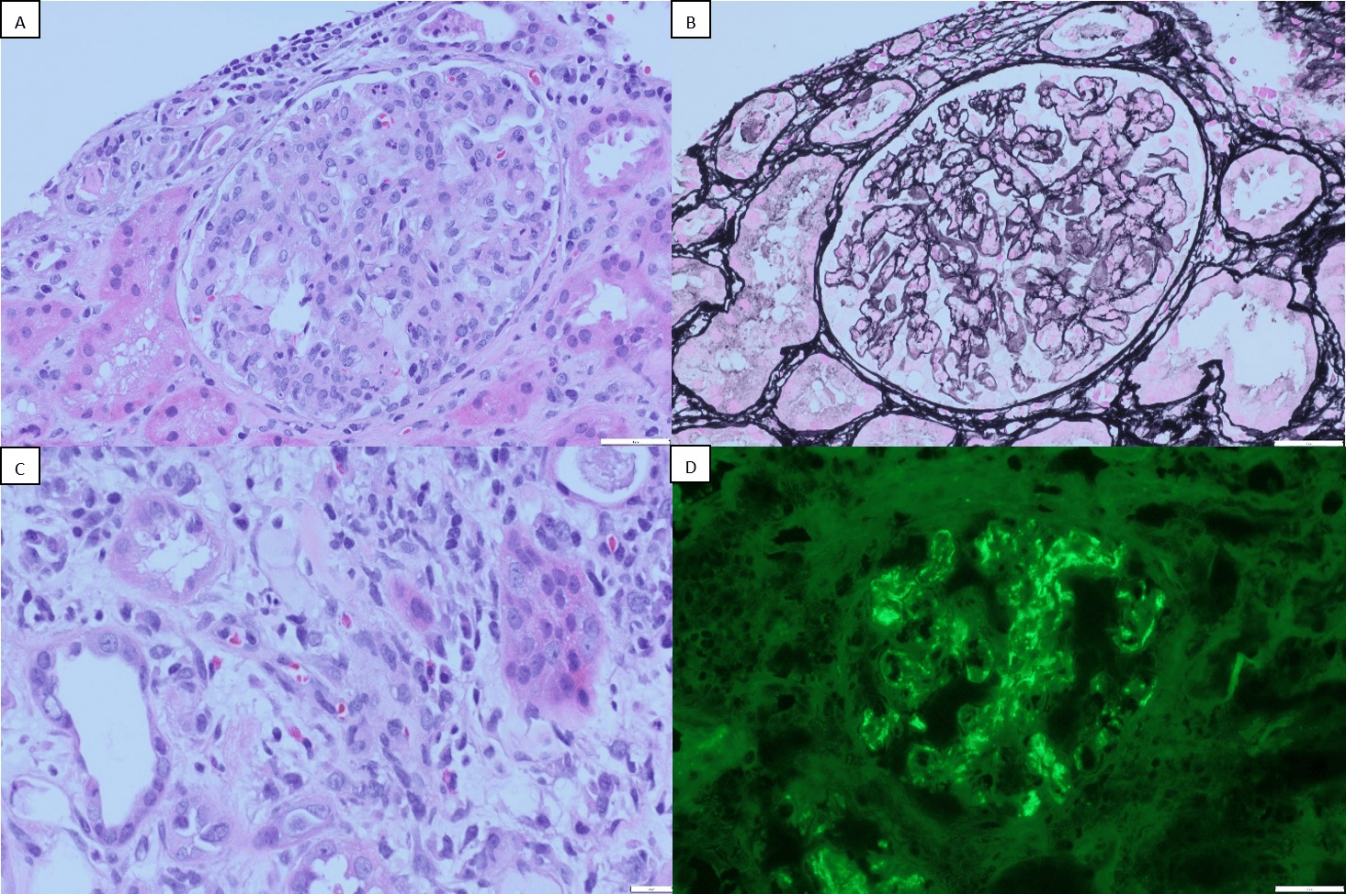
Light microscopy and immunofluorescence findings of the kidney biopsy. A. Hematoxylin and Eosin (HE) staining showing mesangial and endocapillary hypercellularity B.   Jones methenamine silver stain demonstrating endocapillary hypercellularity C. HE staining showing several eosinophils D. Immunofluorescence images showing strong diffuse mesangial C3 deposits

Percutaneous kidney biopsy on hospitalization day 13 demonstrated mesangioproliferative glomerulonephritis with endocapillary hypercellularity in 19 glomeruli (1 globally sclerotic), without crescents, fibrinoid necrosis, or double-contour basement membranes (Figures 4A, 4B). The interstitium contained edema and a moderate inflammatory infiltrate of lymphocytes, plasma cells, occasional neutrophils, and up to 10 eosinophils/hpf (Figure 4C). Immunofluorescence revealed diffuse mesangial C3-dominant deposits (3+) with trace IgG, IgA, C1q, and 1+ IgM; light albumin labeling and kappa/lambda cast staining were also seen (Figure 4D). Electron microscopy did not identify subepithelial “humps”.

**Figure 5:**
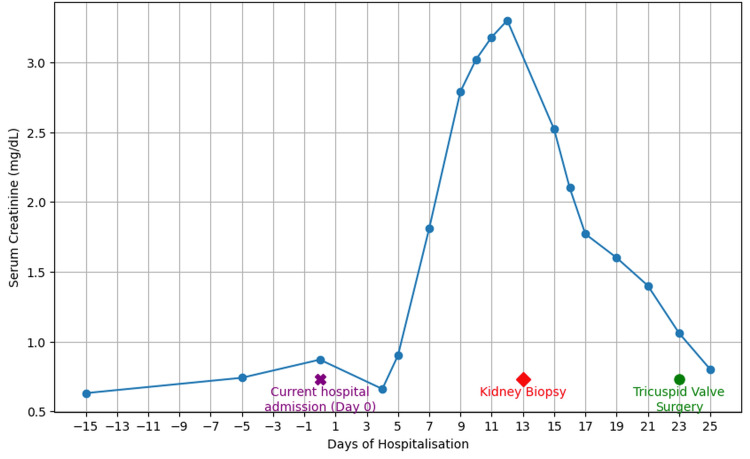
Trend of serum creatinine levels during antibiotic therapy.

Renal function improved with antibiotics and supportive care, serum creatinine levels normalized by day 23 (Figure 5), and the C3 level normalized to 183 mg/d. Tricuspid valve replacement surgery was performed on hospitalization day 23. The patient tolerated surgery well, and serum creatinine level remained within normal limits, without recurrence of AKI in the perioperative period. However, proteinuria persisted and increased from 1.1 to 2.2 g/g, measured on postoperative day 2. Given the patient’s insurance, post-discharge follow-up will continue through the Veterans Affairs clinic for continuity of care.

## Discussion

In cases of AKI in patients with complicated MRSA infective endocarditis, kidney biopsy remains the gold standard for establishing the underlying pathology. Renal involvement is not uncommon in infective endocarditis, and while treatment often focuses on infection control, histologic evaluation is essential to define the cause of AKI. In a young patient with a progressive rise in serum creatinine, it is critical to identify potential contributors such as vancomycin exposure and untreated HCV infection. Possible etiologies include drug-induced nephrotoxicity, HCV-related cryoglobulinemia, interstitial nephritis, and infection-related glomerulonephritis (IRGN). Delaying biopsy in this setting risks missing an opportunity for targeted management. In our case, the decision to proceed with biopsy was supported by the presence of active HCV infection, which can predispose to cryoglobulinemia, and intermittently elevated vancomycin levels concerning for drug-related nephrotoxicity.

The IVDU epidemic has contributed to increasing rates of hepatitis B and C and bacterial infections, most notably *S. aureus*, which remains a leading cause of soft tissue infection, endocarditis, and bacteremia. Currently, most endocarditis-associated glomerulonephritis (GN) identified on kidney biopsy is secondary to acute bacterial endocarditis, with *S. aureus* having replaced *Streptococcus viridans* as the most frequent pathogen. AKI is reported in more than 80% of cases, and low serum complement (mainly C3) is observed in up to 60% of patients. Autoimmune serologies are often positive, including antineutrophil cytoplasmic antibodies (18-33%), antinuclear antibodies, mixed cryoglobulins, rheumatoid factor, and antiphospholipid antibodies [9,10]. Histologically, endocarditis-associated GN demonstrates endocapillary hypercellularity, often with an exudative pattern. On immunofluorescence, C3 is the predominant deposit, with or without IgG or IgA. Electron microscopy typically reveals small mesangial and capillary wall immune deposits, whereas subepithelial humps are identified in fewer than 20% of cases [3,9,11].

The spread of MRSA among IVDU populations has been well documented in the United States. In *S. aureus*-associated GN, cationic bacterial antigens localize in glomeruli, triggering in situ immune complex formation or deposition of circulating immune complexes. This activates complement, generates C3a and C5a, and recruits neutrophils, leading to glomerular injury [12]. Additional mechanisms, such as plasmin activation and host complement dysregulation, may further amplify injury [13]. Although right-sided MRSA endocarditis in injection drug users is well recognized, biopsy-confirmed immune complex-mediated GN in this context is rarely reported.

The cornerstone of management for infective endocarditis-associated GN (IEGN) is prompt, culture-directed antibiotic therapy, with surgical intervention in selected cases. Delayed therapy is associated with poorer renal outcomes. Immunosuppressive treatment has been reported to benefit a subset of patients with persistent renal dysfunction, but its role remains controversial and should be considered only after effective antimicrobial therapy [14]. Despite treatment, 4-33% of adults with IEGN progress to end-stage renal disease. The best outcomes are observed in younger patients without major comorbidities [15,16].

In our case, renal biopsy demonstrated C3-dominant mesangial deposits. Although isolated C3 positivity without IgG, IgM, IgA, or C1q is a diagnostic criterion for C3 glomerulonephritis (C3GN), the patient’s history of MRSA endocarditis and complete recovery with antibiotic therapy support a diagnosis of C3-dominant endocarditis-associated GN. Histologically, IRGN with isolated C3 deposition may be difficult to distinguish from primary C3GN but can occur in up to 25% of IRGN cases, particularly during the resolving or subacute phase [17]. In postinfectious cases, hypocomplementemia and proteinuria typically normalize over time, and on electron microscopy, subepithelial deposits localized to the mesangial “waist” with signs of resorption further support an IRGN diagnosis [18].

Although renal function improved in this patient, proteinuria persisted. Initial proteinuria was 1.1 g/day, and the follow-up urine protein-to-creatinine ratio was 2.2 g/g. The initial urinalysis showed hematuria (3+ blood, >100 RBC/hpf), 1+ proteinuria, cellular casts, and rare urinary eosinophils. Subsequent analysis revealed only trace proteinuria and no casts. The patient was discharged with stable creatinine and scheduled for nephrology follow-up to monitor renal recovery and proteinuria trends after completion of antibiotic therapy and cardiac surgery.

Management strategies may include renin-angiotensin-aldosterone system (RAAS) blockade with angiotensin-converting enzyme (ACE) inhibitors or angiotensin II receptor blockers (ARBs) and possibly sodium-glucose cotransporter 2 (SGLT2) inhibitors to reduce proteinuria and preserve long-term kidney function. Although infection was the apparent trigger, genetic testing for complement pathway abnormalities may be considered if proteinuria persists, as underlying complement dysregulation can predispose to recurrent or persistent glomerular injury. Identifying such abnormalities could guide long-term risk stratification and monitoring [19].

## Conclusions

This case underscores the crucial role of renal biopsy in evaluating complex AKI in the setting of MRSA infective endocarditis, intravenous drug use, and untreated HCV infection. In selected cases, renal biopsy can be safely performed even during active MRSA infection, providing critical diagnostic information to distinguish among potential etiologies such as drug-induced nephrotoxicity, HCV-associated glomerular disease, or primary glomerulopathies that may warrant immunosuppressive therapy. In our case, the biopsy findings-endocapillary hypercellularity with eosinophils and strong, diffuse mesangial C3 deposits-were consistent with IRGN. The reduction in serum creatinine following antibiotic therapy further supported IRGN secondary to *S. aureus *infection. However, proteinuria persisted, and the patient was scheduled for nephrology outpatient follow-up to monitor renal function and proteinuria trends.

IRGN should be considered in patients presenting with AKI, active endocarditis, hematuria, and hypocomplementemia. Although C3-dominant immunofluorescence is characteristic of C3 glomerulopathy, it is not pathognomonic and must be interpreted in the appropriate clinical context. Early, infection-targeted antibiotic therapy guided by timely diagnosis can lead to renal recovery without immunosuppression, while histologic evaluation helps avoid unnecessary immunosuppressive treatment. Overall, this case highlights how the integration of prompt renal biopsy, comprehensive clinical assessment, and etiology-specific therapy can optimize outcomes in patients with infection-associated kidney injury.

## References

[1] Rodriguez-Iturbe B (2021). Autoimmunity in acute poststreptococcal GN: a neglected aspect of the disease. J Am Soc Nephrol.

[2] Iyengar A, Kamath N, Radhakrishnan J, Estebanez BT (2023). Infection-related glomerulonephritis in children and adults. Semin Nephrol.

[3] Satoskar AA, Parikh SV, Nadasdy T (2020). Epidemiology, pathogenesis, treatment and outcomes of infection-associated glomerulonephritis. Nat Rev Nephrol.

[4] Kanjanabuch T, Kittikowit W, Eiam-Ong S (2009). An update on acute postinfectious glomerulonephritis worldwide. Nat Rev Nephrol.

[5] Carapetis JR, Steer AC, Mulholland EK, Weber M (2005). The global burden of group A streptococcal diseases. Lancet Infect Dis.

[6] Nasr SH, Radhakrishnan J, D'Agati VD (2013). Bacterial infection-related glomerulonephritis in adults. Kidney Int.

[7] Nasr SH, Markowitz GS, Stokes MB, Said SM, Valeri AM, D'Agati VD (2008). Acute postinfectious glomerulonephritis in the modern era: experience with 86 adults and review of the literature. Medicine (Baltimore).

[8] Nasr SH, Fidler ME, Valeri AM (2011). Postinfectious glomerulonephritis in the elderly. J Am Soc Nephrol.

[9] Boils CL, Nasr SH, Walker PD, Couser WG, Larsen CP (2015). Update on endocarditis-associated glomerulonephritis. Kidney Int.

[10] Langlois V, Lesourd A, Girszyn N, Ménard JF, Levesque H, Caron F, Marie I (2016). Antineutrophil cytoplasmic antibodies associated with infective endocarditis. Medicine (Baltimore).

[11] Satoskar AA, Suleiman S, Ayoub I (2017). Staphylococcus infection-associated GN - spectrum of IgA staining and prevalence of ANCA in a single-center cohort. Clin J Am Soc Nephrol.

[12] Yousif Y, Okada K, Batsford S, Vogt A (1996). Induction of glomerulonephritis in rats with staphylococcal phosphatase: new aspects in post-infectious ICGN. Kidney Int.

[13] Bokarewa MI, Jin T, Tarkowski A (2006). Staphylococcus aureus: staphylokinase. Int J Biochem Cell Biol.

[14] Bele D, Kojc N, Perše M, Černe Čerček A, Lindič J, Aleš Rigler A, Večerić-Haler Ž (2020). Diagnostic and treatment challenge of unrecognized subacute bacterial endocarditis associated with ANCA-PR3 positive immunocomplex glomerulonephritis: a case report and literature review. BMC Nephrol.

[15] Moroni G, Pozzi C, Quaglini S (2002). Long-term prognosis of diffuse proliferative glomerulonephritis associated with infection in adults. Nephrol Dial Transplant.

[16] Luo C, Tang Z, Chen D, Liu Z (2011). Long-term prognosis for Chinese adult patients with acute postinfectious glomerulonephritis. Clin Nephrol.

[17] Sethi S, Nester CM, Smith RJ (2012). Membranoproliferative glomerulonephritis and C3 glomerulopathy: resolving the confusion. Kidney Int.

[18] Bashir S, Hussain M, Afzal A, Hassan U, Hameed M, Mushtaq S (2021). C4d at crossroads between post-infectious glomerulonephritis and C3 glomerulopathy. Int J Nephrol Renovasc Dis.

[19] Schena FP, Esposito P, Rossini M (2020). A narrative review on C3 glomerulopathy: a rare renal disease. Int J Mol Sci.

